# Supplementary material for: Structural Equation Modeling of Common Cognitive Abilities in Preschool-Aged Children Using WPPSI-IV and BRIEF-P
Source: Children (Basel). 2022 Jul 21;9(7):1089. doi: 10.3390/children9071089 (PMC9323403; doi:10.3390/children9071089)
Supplement: Supplementary file 1 [file children-09-01089-s001.zip › children-1679110-supplementary.pdf]

Supplementary Materials:

Table S1. Descriptive values for BRIEF-P Scales and WPPSI-IV indices and fullscales.

**Indices WPPSI-IV**	**M**	**SD**	**min.**	**max.**	**< 85 n (%)**	**> 115 n (%)**
VCI	102.65	14.984	66	132		
VSI	107.51	14.815	58	148		
FRI	106.40	16.003	74	145		
WMI	101.50	15.024	67	146		
PSI	99.83	12.281	66	130		
FSIQ	104.94	14.341	71	138	12 (9.7)	30 (24.2)
VAI	100.74	12.956	70	127		
NVI	105.69	15.277	73	149		
GAI	105.49	14.226	69	141		
CPI	101.02	14.338	65	137		
BRIEF-P Scales	M	SD	min.	max.	> 65 n	percent
WM	48.40	9.369	37	80	9	7.3
INH	48.56	8.846	36	72	8	6.5
SH	49.12	9.524	37	78	11	8.9
EC	47.22	9.297	35	75	5	4.0
PO	48.57	10.190	33	80	7	5.6
GEC	48.03	9.352	33	77	8	6.5

Note. VCI—Verbal Comprehension Index, VS—Visual Spatial Index, FRI—Fluid Reasoning Index, WM—Working Memory Index, PSI—Processing Speed Index, FSIQ—Fullscale-IQ; Secondary Indices: VAI—Verbal Acquisition Index, NVI—Nonverbal Index, GAI—General Ability Index, CPI—Cognitive Proficiency Index; WM—Working Memory, INH—Inhibit, SH—Shift, EC= Emotional Control, PO—Plan/Organize, GEC—Global Executive Composite Functioning.

Table S2. Descriptive statistics of the indicators within the sample (N = 124).

	**M**	**SD**	**min**	**max**	**Kurtosis**	**c.r.**
Information	10.27	2.852	2	18	0.535	1.197
Similarities	10.73	3.137	2	17	-0.103	-0.231
Receptiv Vocabulary	10.57	2.777	5	16	-0.752	-1.682
Picture Naming	9.76	2.748	4	17	-0.300	-0.671
Picture Memory	10.21	3.264	2	19	0.175	0.392
Zoo Location	10.27	2.795	2	18	0.760	1.700

Block Design	11.24	3.181	3	19	0.193	0.430
Object Assembly	11.23	2.816	2	17	0.673	1.506
Matrix Reasoning	11.35	3.092	3	18	-0.317	-0.709
Picture Concepts	10.85	3.311	3	17	-0.264	-0.589
Bug Search	10.46	2.484	3	17	0.947	2.117
Cancellation	9.40	2.572	1	16	0.635	1.420
Working Memory	48.40	9.369	37	80	0.374	0.837
Inhibition	48.56	8.846	36	72	-0.061	-0.136
Shift	49.12	9.524	37	78	0.449	1.005
Emotional Control	47.22	9.297	35	75	0.243	0.544
Plan/ Organization	48.57	10.190	33	80	0.352	0.788
Multivariate					5.171	1.114

Note. Abbreviations of subtests: IN—Information; SI—Similarities; RV—Receptive Vocabulary; PN—Picture Naming; BD—Block Design; OA—Object Assembly; MR—Matrix Reasoning; PCs—Picture Concepts; PM—Picture Memory; ZLs—Zoo Locations; BS—Bug Search; CA—Cancellation.

Table S3. Correlation of WPPSI-IV Indices and fullscale IQ and BRIEF-P scales and GEC (N = 124).

**Indices**	VCI	VSI	FRI	WMI	PSI	FSIQ	WM	INH	SH	EC	PO
VSI	0.321**	--									
FRI	0.446**	0.505**	--								
WMI	0.279**	0.391**	0.462**	--							
PSI	0.334**	0.421**	0.449**	0.303**	--						
FSIQ	0.748**	0.666**	0.754**	0.608**	0.573**	--					
WM	-0.231**	-0.242**	-0.293**	0.,110	-0.307**	-0.253**	--				
INH	-0.197*	-0.142	-0.310**	-0.132	-0.285**	-0.230**	0.656**	--			
SH	-0.168*	-0.109	-0.148	-0.018	-0.039	-0.105	0.388**	0.325**	--		
EC	-0.141	-0.127	-0.159*	-0.101	-0.176*	-0.126	0.468**	0.620**	0.540**	--	
PO	-0.179*	-0.022	-0.141	-0.039	-0.171*	-0.116	0.762**	0.626**	0.255**	0.438**	--
GEC	-0.243**	-0.157*	-0.287**	-0.119	-0.258**	-0.226**	0.859**	0.859**	0.558**	0.746**	0.784**

Note. Spearman-Rho; **. Correlation is sig. 0.01 level (one-sided); *. Correlation sig. 0.05 level (one-sided).

Table S4. Correlation of WPPSI-IV subtest and BRIEF-P scales (N = 124).

**.**	**IN**	**SI**	**RV**	**PN**	**BD**	**OA**	**MR**	**PC**	**PM**	**ZL**	**BS**	**CA**	**WM**	**INH**	**SH**	**EC**
SI	0.625**	--														

RV	0.372**	0.387**	--													
PN	0.483**	0.433**	0.405**	--												
BD	0.200*	0.276**	0.111	0.101	--											
OA	0.181*	0.267**	0.153*	0.066	0.417**	--										
MR	0.268**	0.294**	0.159*	0.209*	0.516**	0.296**	--									
PC	0.370**	0.503**	0.324**	0.226**	0.455**	0.286**	0.408**	--								
PM	0.288**	0.258**	0.116	0.129	0.280**	0.252**	0.224**	0.330**	--							
ZL	0.147	0.172*	0.217**	0.064	0.311**	0.262**	0.395**	0.304**	0.357**	--						
BS	0.225**	0.314**	0.199*	0.043	0.385**	0.211**	0.360**	0.396**	0.206*	0.297**	--					
CA	0.167*	0.368**	0.201*	0.095	0.290**	0.304**	0.183*	0.372**	0.168*	0.202*	0.436**	--				
WM	-0.175*	-0.219**	-0.379**	-0.183*	-0.218**	-0.192*	-0.132	-0.395**	-0.100	-0.089	-0.197*	-0.341**	--			
INH	-0.146	-0.205*	-0.341**	-0.054	-0.121	-0.112	-0.184*	-0.336**	-0.095	-0.112	-0.227**	-0.253**	0.656**	--		
SH	-0.109	-0.185*	-0.162*	-0.166*	-0.088	-0.087	-0.065	-0.207*	0.039	-0.103	-0.012	-0.129	0.388**	0.325**	--	
EC	-0.045	-0.208*	-0.153*	-0.056	-0.077	-0.127	-0.053	-0.224**	-0.022	-0.167*	-0.113	-0.213**	0.468**	0.620**	0.540**	--
PO	-0.166*	-0.136	-0.249**	-0.133	-0.048	0.012	-0.006	-0.243**	-0.063	-0.027	-0.044	-0.272**	0.762**	0.626**	0.255**	0.438**

Note. Spearman-Rho; **. Correlation is sig. 0.01 level (one-sided); *. Correlation sig. 0.05 level (one-sided).

Table S5. Comparison of estimated parameters of structural relationships with and without control of common method variance.

Structural relationships explanatory variable (g)	Model without method factor		Model with method factor	
	SRW	SMC	SRW	SMC
VCI	0.640		0.999*	
VSI	0.850		0.605	
FRI	0.998*		0.991*	
WMI	0.794		0.441	
PSI	0.817		0.528	
GEC	-0.427	0.182	-0.272	0.074
Varinance of g	0.188	p=0.020	0.636	p= 0.000

Note: SRW—Standardized regression weights; SMC—Squared multiple correlation;* Fixation of error variances to smallest value 0.001.

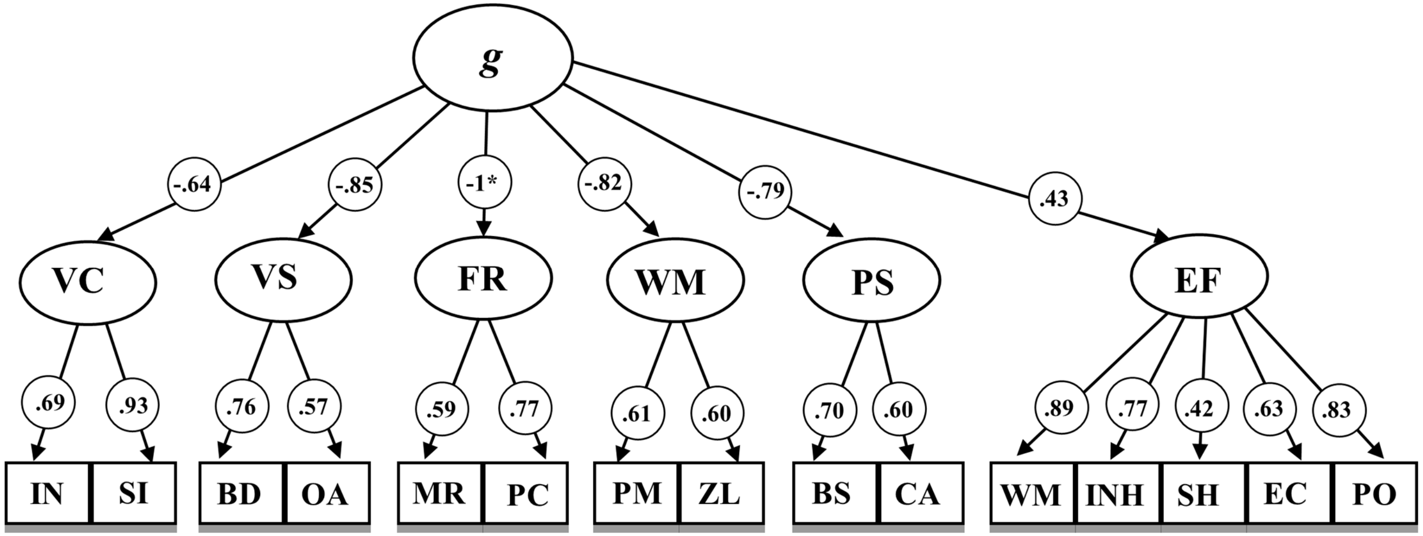

χ^2 = 144.419, df = 85, p = 0.001; CFI = 0.908; RMSEA = 0.075

Figure S1. Second-order six-factor model including standardized estimations for the CFA_1b (N = 124) of the ten WPPSI-IV subtests and five BRIEF-P scales (CFA_1 in Table 4).

Note. CFA—confirmatory factor analysis; WPPSI-IV—Wechsler Primary and Preschool Scale of Intelligence – fourth edition; IN—Information, SI—Similarities, CO—Comprehension, RV—Receptive Vocabulary, PN—Picture Naming; BD—Block Design, OA—Object Assembly, MR—Matrix Reasoning, PC—Picture Concepts, PM—Picture Memory, ZL—Zoo Locations, BS—Bug Search, CA—Cancellation, VC—Verbal Comprehension, VS—Visual Spatial, FR—Fluid Reasoning, WM—Working Memory, PS—Processing Speed, g—General Intelligence,; BRIEF-P scales: WM= Working Memory, INH= Inhibit , SH= Shift, EC= Emotional Control, PO= Plan/Organize, EF= Fullscale Executive Functions;* Fixation of error variances to smallest value 0.001. All standardized parameter estimates are significant at p < 0.001.

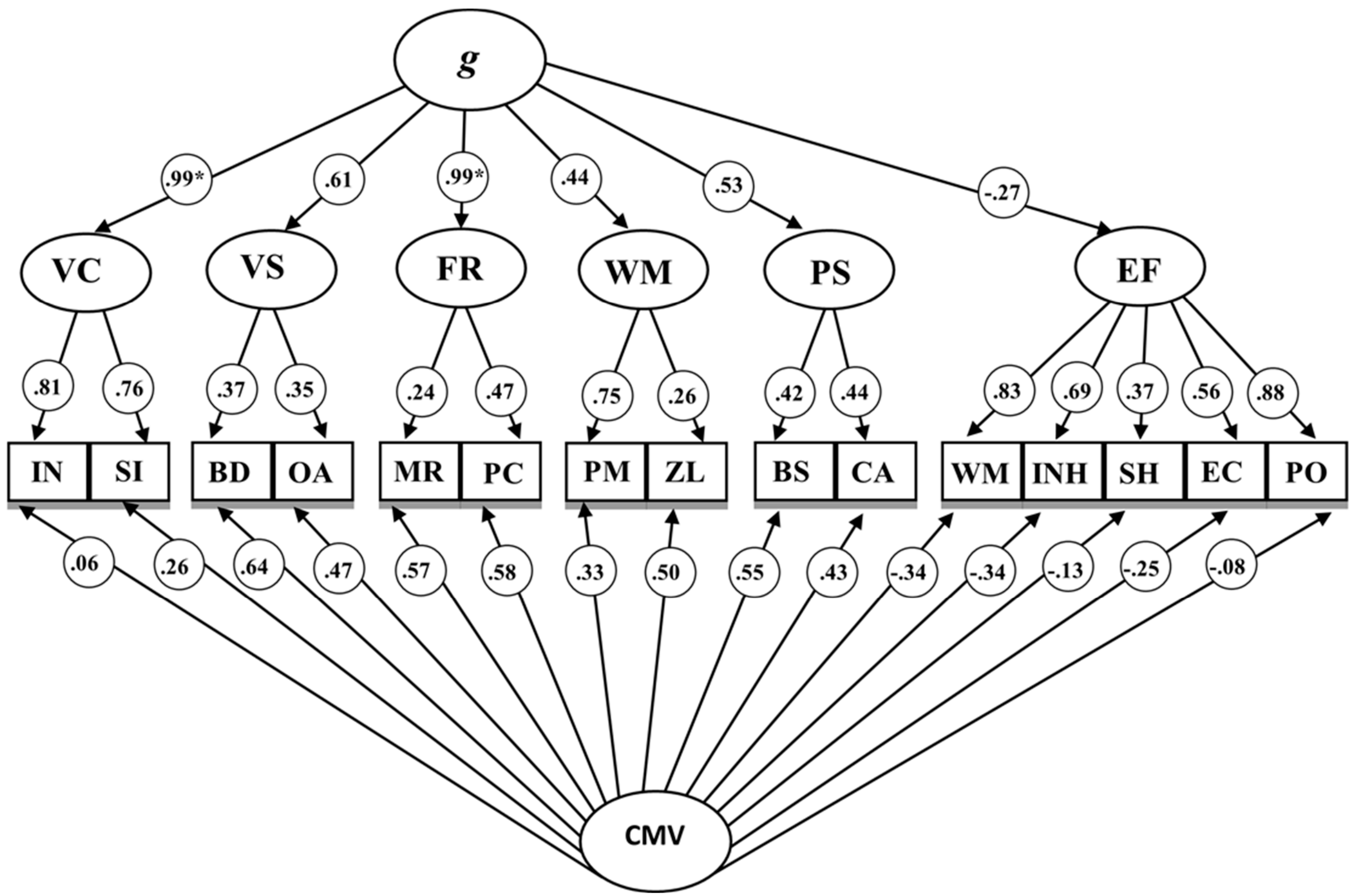

$\chi^2 = 120.498$, df = 71, p = 0.001; CFI = 0.923; RMSEA = 0.075

Figure S2. Second-order six-factor model including a primary latent method factor, with standardized estimations for the CFA_cmv, (N = 124) of the ten WPPSI-IV subtests and five BRIEF-P scales (CFA_1 in Table 5).

Note. CFA—Confirmatory factor analysis; WPPSI-IV—Wechsler Primary and Preschool Scale of Intelligence – fourth edition; IN—Information, SI—Similarities, CO—Comprehension, RV—Receptive Vocabulary, PN—Picture Naming; BD—Block Design, OA—Object Assembly, MR—Matrix Reasoning, PC—Picture Concepts, PM—Picture Memory, ZL—Zoo Locations, BS—Bug Search, CA—Cancellation, VC—Verbal Comprehension, VS—Visual Spatial, FR—Fluid Reasoning, WM—Working Memory, PS—Processing Speed, *g*—General Intelligence; BRIEF-P scales: WM= Working Memory, INH= Inhibit , SH= Shift, EC= Emotional Control, PO= Plan/Organize, *f*EF= Fullscale Executive Functioning; * Fixation of error variances to smallest value 0.001. All standardized parameter estimates are significant at $p < 0.001$.

Table S6. Reliability of the final measurement model.

	vci		vsi		fri		wmi		psi		EF				
	**IN**	**SI**	**BD**	**OA**	**MR**	**PC**	PM	ZL	BS	CA	WM	INH	SH	EC	PO
Factor reliability	0.863		0.728		0.792		0.719		0.725		0.869				
AVE	0.759		0.579		0.656		0.564		0.572		0.582				
IIC	0.639		0.429		0.421		0.364		0.420		0.512				
α	0.780		0.600		0.592		0.534		0.592		0.840				
Indicator reliabily	0.699	0.819	0.76	0.397	0.621	0.691	0.679	0.449	0.711	0.434	0.85	0.653	0.217	0.453	0.736
Factor loading	0.836	0.905	0.872	0.63	0.788	0.831	0.824	0.67	0.843	0.659	0.922	0.808	0.466	0.673	0.858

Note. N = 124, AVE—Average variance extracted; IIC—Inter-Item Correlation, Abbreviations of factors: vci—Verbal Comprehension Index , vsi—Visual Spatial Index , fri—Fluid Reasoning Index, wmi—Working Memory Index, psi—Processing Speed Index , EF—Executive Function; Abbreviations of subtests of the WPPSI-IV: : IN—Information; SI—Similarities; RV—Receptive Vocabulary; PN—Picture Naming; BD—Block Design; OA—Object Assembly; MR—Matrix Reasoning; PCs—Picture Concepts; PM—Picture Memory; ZLs—Zoo Locations; BS—Bug Search; CA—Cancelation; Abbreviations of the BRIEF-P scales: WM= Working Memory, INH= Inhibit, SH= Shift, EC= Emotional Control, PO—Plan/Organize.

Table S7. Frequencies of Inconsistency Scale and Negativity Scale of the BRIEF-P..

**value ranges**	**inconsistence frequency**	**percent**	**Neg.-Items**	**negativity frequency**	**percent**
0-5	54	44,1	0	100	81,3
6-10	66	54,2	1	13	10,6
≥ 11	2	1,6	2	7	5,7
			3	1	0,8
			4	2	1,6

Note. Inconsistence scale, n = 122; Negativity scale, n = 123.
